# Multiple Photographs of a Perspective Scene Reveal the Principles of Picture Perception

**DOI:** 10.3390/vision2030026

**Published:** 2018-06-26

**Authors:** Casper J. Erkelens

**Affiliations:** Experimental Psychology, Helmholtz Institute, Utrecht University, Heidelberglaan 1, 3584 CS Utrecht, The Netherlands; c.j.erkelens@uu.nl

**Keywords:** picture perception, pictorial distance, angular size

## Abstract

A picture is a powerful and convenient medium for inducing the illusion that one perceives a three-dimensional scene. The relative invariance of picture perception across viewing positions has aroused the interest of painters, photographers, and visual scientists. This study explores variables that may underlie the invariance. It presents a computational analysis of distances and directions in sets of two photographs of perspective scenes taken from different camera positions. Focal lengths of the lens and picture sizes are chosen such that the sizes of one of the familiar objects are equally large in both photographs. The selected object is perceived at the same distance in both photographs, independent of viewing distance, showing that pictorial distance is fully determined by angular size of the object. Pictorial distance is independent of camera position, focal length of the lens, and picture size. Distances and directions of pictorial objects are computed as a function of viewing distance, and compared with distances and directions of the physical objects as a function of camera position. The computations show that ratios between pictorial distances, directions, and angular sizes of objects in a photograph are constant, as a function of viewing distance. The constant ratios are proposed as the reason for invariance of picture perception over a range of viewing distances. Reanalysis of distance judgments obtained from the literature shows that perspective space, previously proposed as the model for visual space, is also a good model for pictorial space. The geometry of pictorial space contradicts some conceptions about picture perception.

## 1. Introduction

Pictures are images on flat surfaces, in which human subjects can see objects at a distance (i.e., relative to the viewer) and in depth (i.e., relative to other objects). Defined in this way, pictures are both two-dimensional objects (e.g., made of canvas, paper, or pixels) and planar representations of three-dimensional scenes (e.g., landscapes or portraits). A convenient aspect of pictures is their viewpoint-independent utility. That is, a viewer need not be directly in front of a picture at the point from which it was taken to enjoy it, to understand it, to admire it, or simply to look at it and make sense of what is seen [1]. Picture perception has been studied during oblique viewing. Many authors concluded that viewers compensate for incorrect viewpoints. They advocated theories of picture perception, relying on mental operations that rectify the Euclidean geometry of the original scene [2,3,4,5,6,7,8,9,10,11]. Busey and colleagues [1] claimed that one can look at moderately slanted pictures without perceptual interference because the distortions in the image are subthreshold, or within the bounds of acceptability. Other authors did not find evidence for viewpoint compensation [12,13,14]. The current computational study explores variables related to distances, directions, and sizes of pictorial objects, which may underlie the relative invariance for incorrect viewing positions.

Generally, picture perception and real-world perception have been conceived as different. Gibson [15], Sedgwick [16], Costall [17], Hagen [18], Hochberg [19,20], Kennedy [21], Kubovy [22], Rogers [23], and Willats [24] emphasized differences between perception of the world and pictures of it. Koenderink and colleagues [25] proposed that pictorial space should not be thought of as “three-dimensional,” but rather as “two-plus-one-dimensional,” the single dimension being “depth”. Pictorial space was described as a fiber space, with the visual field as base space, and the depth dimension as fibers [26]. The description specified that base and fibers have fully distinct geometrical structures (the base space approximately Euclidean, the fibers close to affine) and are largely independent of each other. Koenderink and colleagues [27,28] further argued that familiar size is not a distance cue in picture perception. Wagemans et al. [28] recognize that the size cue is well understood for the perception of physical objects, i.e., for visual space. Familiar size can act as an effective distance cue because the distance from the eye to an object equals the ratio of its physical size to its angular extent in the visual field. Koenderink and colleagues [27,28] argue that such simple geometrical relations do not apply to pictorial space, since the eye itself is not in pictorial space, and consequently the notion “distance from the eye” is meaningless. The eye not being an object in pictorial space is a fallacious argument because it creates an irrelevant distinction between pictorial space and physical space. The relevant distinction to be made is between physical space on the one side, and perceptual spaces, such as visual space and pictorial space, on the other side. The eye is neither an object in visual space, yet, numerous studies showed that distance is a useful concept for judging the remoteness of physical objects [29,30,31,32,33,34,35,36,37,38,39,40,41,42,43,44,45,46,47,48,49,50]. The physical position that most obviously qualifies for being the reference for distance and direction in pictorial space is the position from which the picture is viewed.

In a number of experimental and computational studies, I investigated the geometry of perspective space and proposed it as a model for visual space [51,52,53,54,55,56]. Experiments included judgments on distance and size of physical and depicted objects. Two conclusions relevant for the current study were that (1) familiar shape and size are powerful cues for slant and distance perception, and (2) apart from a stronger underestimation of slant and distance, there was no reason to assume a different geometry for pictorial space. To further test the hypothesis that the geometries of pictorial and visual space are similar, this study presents computations made on sets of two photographs of a perspective scene containing familiar objects. The pictures are taken from different distances. Focal length of the lens and picture size are manipulated such that the sizes of one of the familiar objects become identical in both photographs. These manipulations reveal the effects of focal length and picture size on picture perception. Computation of distances and directions of depicted and physical objects enables comparison between the geometries of pictorial and physical space. Comparison between distances in pictorial space and visual space comes from data in the literature. Data obtained by Kraft and Green [57] of the perceived distance of depicted objects as function of their physical distance is fitted to the perspective model of visual space [56]. The computations of perceived distances and directions in this study are based on the following hypothesis: “When looking at a picture, viewers perceive the distance of a depicted object (the *physical* distal stimulus) as the distance of an imaginary physical object (the *pictorial* distal stimulus) that produces the same retinal image (the *proximal* stimulus)”. The hypothesis proved to be successful in describing perceived slant of obliquely viewed grid figures as functions of depicted slant and slant of the picture [51,52].

## 2. Comparison of Photographs Taken from Different Camera Positions

**Figure 1:**
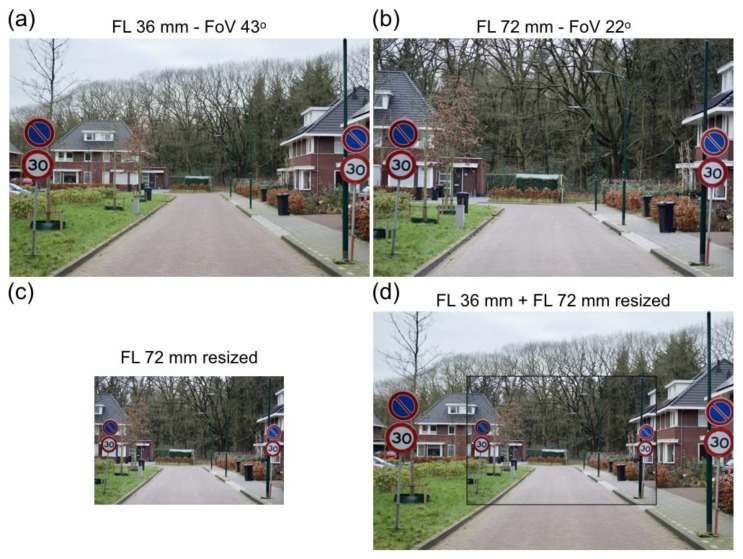
Two pictures of the same perspective scene. (**a**) The picture was taken with a camera equipped with a lens having a focal length (FL) of 36 mm and a field of view (FoV) of 43°. (**b**) The picture was taken with another lens (FL 72 mm, FoV 22°) from another camera position. (**c**) The picture is a resized version of Figure (**b**). The house on the left side is scaled to that of Figure (**a**). (**d**) Figure (**c**) including a thin black rim is placed on top of Figure (**a**).

Figure 1a,b show two photographs of the same perspective scene taken with a digital SLR camera (Nikon D5100). Size of the camera’s APS-C sensor is 15.7 × 23.6 mm, so that a focal length (FL) of 36 mm corresponds with a field of view (FoV), defined as the diagonal angle of view of the lens, of 43°. An FL of 72 mm corresponds with a FoV of 22°. Figure 1a was taken with the FL 36 mm lens and Figure 1b with the FL 72 mm lens. The pictures were taken from two camera positions, such that objects nearby the camera, e.g., the traffic signs, were projected to similar locations in the pictures. By printing both pictures at the same size, the traffic signs have the same size in both pictures. The traffic signs appear at the same distance when you look into the pictures. The traffic signs remain at one distance, irrespective of the viewing distance. Far objects have different sizes in Figure 1a,b. The house at the left side is smaller in Figure 1a than in Figure 1b. The house in Figure 1a is seen at a larger distance than the house in Figure 1b. The difference in distance seems to support the general opinion that lenses of different focal lengths make a scene look compressed or expanded in depth [58]. Short lenses expand depth, whereas long lenses compress depth. A pertinent question is why this is the case. Figure 1c,d show that depth compression is not equivalent to distance compression. Figure 1b has been resized to Figure 1c such that the houses in Figure 1a,c are equal of size. Figure 1c is a factor of 1.92 smaller than Figure 1a,b. Placement of Figure 1c on Figure 1a, as has been done in Figure 1d, demonstrates that the distant house on the left side is, indeed, equally large in both pictures. The house is also seen at the same distance in both pictures. Reducing house size increases perceived distance. The traffic signs are smaller and perceived at a longer distance in the small pictures. Equally perceived distances in Figure 1c,d show that picture size, per se, is irrelevant for distance perception. Depicted object size seems the factor that determines perceived distance, not picture size. Changing size changes perceived distances but not depth. Figure 1d shows that depth between traffic signs and house is still compressed in the FL 72 mm picture relative to that in the FL 36 mm picture. Computations in the next paragraphs will reveal the distance information that characterizes depth.

## 3. Geometry of the Physical Scene

**Figure 2:**
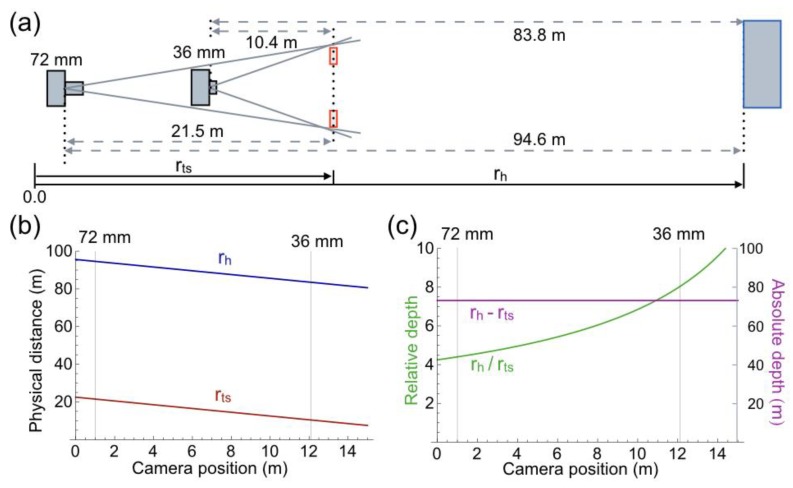
Geometry of the physical scene depicted in Figure 1a,b. (**a**) Top view of the scene. Sizes and distances of objects are not to scale. Distances were computed of traffic signs (r_ts_) and house (r_h_) relative to the positions from which the pictures were taken. (**b**) Distance of traffic signs (r_ts_) and house (r_h_) as function of camera position. The arbitrary origin of the x-axis is chosen 1 m from the FL 72 mm camera position. Thin vertical lines mark the two camera positions from which the pictures were taken. (**c**) Relative (green) and absolute (magenta) depth between traffic signs and house computed as function of camera position.

Distances of traffic signs and house, relative to the camera positions, were computed from the angular sizes of traffic signs and house in the pictures. Computations were made on pictures of Figure 1a,b measuring 8.9 × 13.5 cm. Knowledge of the FoVs of the two pictures and physical size of one familiar object was a prerequisite for the unambiguous computation of the various distances. The 30 km traffic sign at the left side of the road, having a physical diameter of 60 cm, served as the familiar object in the computations. Figure 2a shows the distances computed from the pictures. The computed distances were verified by measuring the actual distances in physical space. Errors are smaller than 2%. The computed height of the house is 10.44 m, while it actually is 10.23 m (data supplied by the builder of the houses). Figure 2b shows distances of traffic sign and house as function of camera position. The distances enable the computation of two measures of depth. Absolute depth is defined as the difference between distances of the house and traffic sign. Relative depth is defined as the ratio between distances of the house and traffic sign. Since the traffic signs and house are stationary objects, absolute depth is independent of camera position (Figure 2c). Relative depth increases exponentially with more forward camera positions, until it reaches infinity, when the camera passes the traffic signs. The ratio is 4.4 at the position of the FL 72 mm picture, and 8.1 at the position of the FL 36 mm picture. Distances and depths in physical space were computed in order to compare them to similar measures in pictorial space in the next paragraph.

## 4. Geometry of the Pictorial Scene

### 4.1. Distance

**Figure 3:**
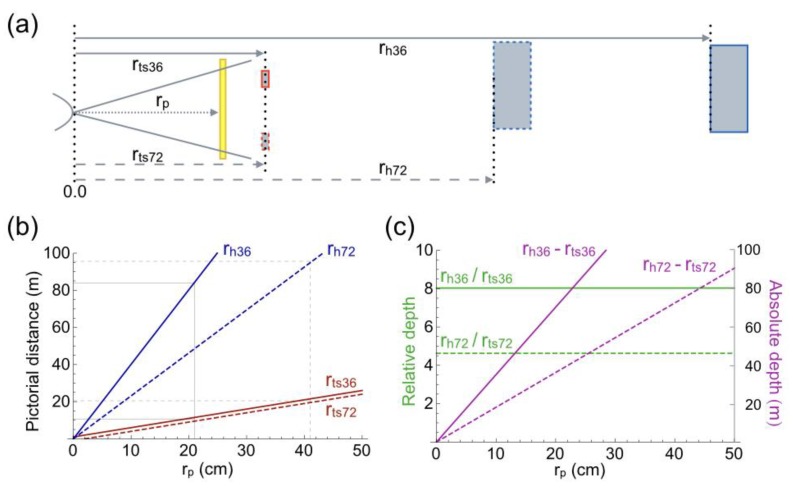
Geometry of the pictorial scenes of Figure 1a,b. (**a**) Top view of the pictorial scene. Sizes and distances of objects are not to scale. Distances were computed for the traffic signs (r_ts36_ and r_ts72_) and house (r_h36_ and r_h72_) depicted in the two pictures. The viewer (curve at the left side) is looking at the pictures (yellow bar) from a distance r_p_. (**b**) Pictorial distances of traffic signs and houses that produce the same retinal images as their projections in the pictures. Distances are computed as function of r_p_. Thin lines mark the viewing distances at which the computed distances are equal to the physical distances (see Figure 2a). (**c**) Relative (green) and absolute (magenta) depth between the computed distances of traffic signs and houses as function of r_p_.

First, a few observations are made about the perceived distances of objects in the pictures of Figure 1a,b. The traffic signs are perceived at the same distance (r_ts36_ and r_ts72_) in both pictures, irrespective of the viewing distance (r_p_). Common properties of the traffic signs are equal angular size in the pictures and equal size in physical space. The house is seen at the longest distance (r_h36_) in Figure 1a, and at a shorter distance (r_h72_) in Figure 1b. Depth between traffic sign and house is compressed in Figure 1b relative to Figure 1a. The compression remains present for other viewing distances. For each viewing distance r_p_, pictorial distances of traffic sign and house were computed from their size in physical space and their angular size in the pictures. Figure 3a shows, qualitatively, the geometry of the pictorial scene. Figure 3b shows that pictorial distances increase linearly with viewing distance r_p_. The thin solid lines in Figure 3b indicate that pictorial distances are equal to physical distances at a viewing distance of 21 cm for the FL 36 mm picture, whereas this equality occurs at a viewing distance of 41 cm for the FL 72 mm picture. Depth is compressed for shorter viewing distances and expanded for longer viewing distances. The observation that the traffic signs are perceived at the same distance in both pictures shows that knowledge of camera positions and focal lengths are irrelevant for pictorial distance. Pictorial distance is determined by the angular size of the depicted object and knowledge of the object’s size in physical space. Figure 3c shows computations of depth. Absolute depth increases linearly with viewing distance (Figure 3c). The slope is 48% steeper for the FL 36 mm picture. Relative depth is constant, as a function of viewing distance_._ The ratio is 8.1 for the FL 36 mm picture and 4.4 for the FL 72 mm picture, which is a difference of 42%. Both types of depth seem consistent with the compression of depth perceived in the FL 72 mm picture of Figure 1b relative to that in the FL 36 mm picture of Figure 1a, independent of viewing distance.

**Figure 4:**
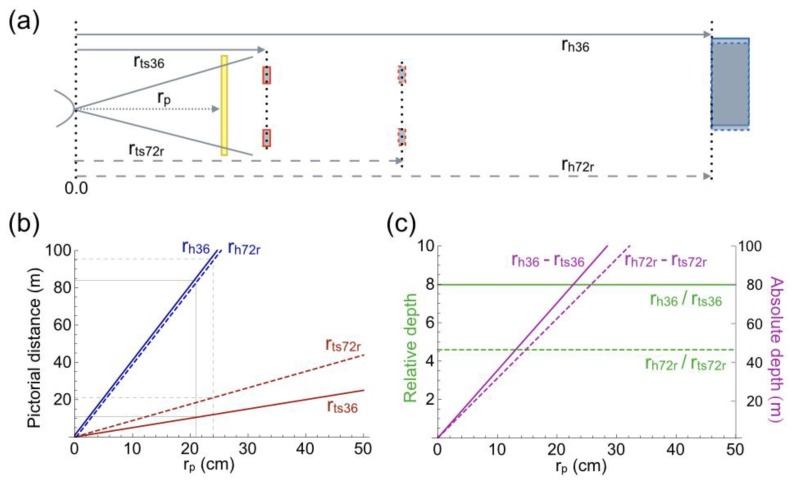
Geometry of the pictorial scenes of Figure 1d. (**a**) Top view of the pictorial scene. Sizes and distances of objects are not to scale. Distances were computed for the traffic sign (r_ts36_ and r_ts72r_) and house (r_h36_ and r_h72r_) depicted in the two pictures. The viewer (curve at the left side) is looking at the pictures (yellow bar) from a distance r_p_. (**b**) Pictorial distances of traffic signs and houses that produce the same retinal images as their projections in the pictures. Distances are computed as function of r_p_. Thin lines mark the viewing distances at which the computed distances are equal to the physical distances (see Figure 2a). (**c**) Relative (green) and absolute (magenta) depth between the computed distances of traffic signs and houses as function of r_p_.

Further evidence for the thesis that angular size specifies pictorial distance comes from the small pictures of Figure 1c,d, where Figure 1c has been placed on top of Figure 1a. Both traffic sign and house appear further away in Figure 1c,d than in Figure 1b. The house in Figure 1a,d appears at the same distance. Figure 4a shows, qualitatively, the geometry of the pictorial scene of Figure 1d. Figure 4b,c show the computed pictorial distances. Figure 4b shows that the two traffic signs have very different distances now, whereas the distances of the houses are, indeed, equal. The equal distances of the house show again that pictorial distance is determined by the angular size of the depicted object and knowledge of the object’s size in physical space. The thin solid lines in Figure 4b indicate that pictorial distances are equal to physical distances at a viewing distance of 21 cm for the FL 36 mm picture, whereas this equality occurs at a viewing distance of 24 cm for the small FL 72 mm picture. The difference between the two viewing distances reflects the fact that the two pictures were taken from different camera positions (Figure 2a). Resizing Figure 1b to Figure 1c changes absolute depth, but not relative depth (compare Figure 3c and Figure 4c). Absolute depth increases linearly with viewing distance again (Figure 4c). Reduction in size of the FL 72 mm picture has increased absolute depth to a level that approaches absolute depth in the FL 36 mm picture. The difference in slope is reduced to just 12%. Reduction in size of the FL 72 mm picture does not have any effect on relative depth. Relative depth is independent of picture size. The independence of picture size seems compatible with the depth compression that is perceived between house and traffic sign in the large (Figure 1b) and small (Figure 1d) FL 72 mm pictures. The next paragraph explores how relative depth is related to angular size.

### 4.2. Angular Size

**Figure 5:**
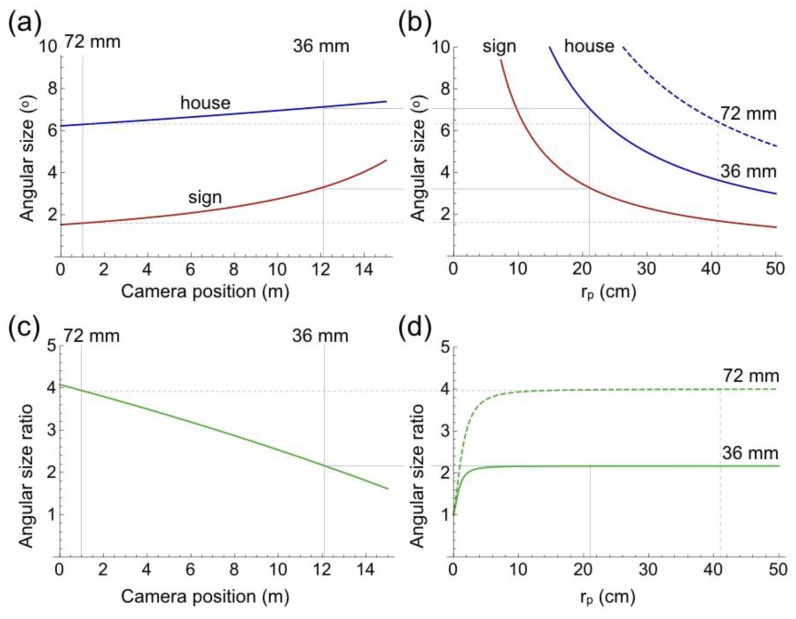
Angular size. (**a**) Angular sizes of the physical traffic sign (red) and house (blue) as function of camera position. The origin of the camera-position scale is given in Figure 2a. (**b**) Angular sizes of the depicted traffic sign (red) and house (blue) as function of viewing distance of the picture (r_p_). The solid blue line shows the angular size of the house in the 36 mm picture of Figure 1a, the dashed blue line that of the house in the 72 mm picture of Figure 1b. (**c**) Ratio between angular sizes of the physical house and traffic signs shown in (**a**). (**d**) Ratio between angular sizes of the depicted house and traffic signs shown in (**b**). The horizontal solid line shows the ratio in the 36 mm picture and the dashed line in the 72 mm picture.

Computation of angular size is relevant because of the demonstrated relationship to perceived distance in pictures. Figure 5a shows angular sizes of the physical traffic sign and house as function of camera position. Angular size of the traffic sign shows much more variation than that of the house, due to the much shorter distance between traffic sign and camera. Variations in the angular sizes are much more similar in pictorial space (Figure 5b), due to almost equal distances of the depicted traffic sign and house to the viewer. Angular size ratio, defined as angular size of the house divided by angular size of the traffic sign, was computed to demonstrate the differences between variations in detail. In physical space, angular size ratio decreases with more forward positions of the camera because the traffic sign increases much faster in size than the house does (Figure 5c). In pictorial space, angular size ratio remains constant for viewing distances longer than 10 cm because the depicted traffic sign and house are equally distant from the viewer (Figure 5d). Magnitude of the constant ratio is determined by the angular sizes of traffic sign and house at the positions from which the photographs are taken (Figure 5a). Focal length of the lens, as such, does not affect the angular size ratios, because changes of focal length amplify the angular sizes of traffic sign and house by identical factors.

### 4.3. Visual Direction

**Figure 6:**
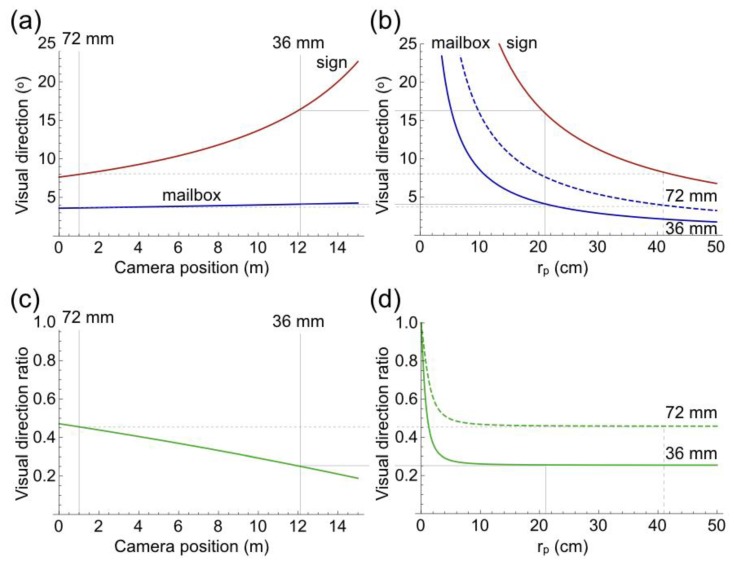
Visual directions. (**a**) Visual directions of the center of the physical 30 km traffic sign standing at the left side of the road (red) and the mailbox of the distant house (blue) as function of camera position. The origin of the camera-position scale is given in Figure 2a. (**b**) Visual directions as function of viewing distance of the picture (r_p_). The solid blue line shows the visual direction of the mailbox in the 36 mm picture of Figure 1a, the dashed blue line, of the mailbox in the 72 mm picture of Figure 1b. Thin lines mark the viewing distances at which the visual directions of projected objects are equal to the visual directions of the physical objects (see Figure 2a). (**c**) Ratio between visual directions of the physical house and traffic signs shown in (**a**). (**d**) Ratio between visual directions of the depicted house and traffic signs shown in (**b**). The solid line shows the ratio in the 36 mm picture and the dashed line in the 72 mm picture.

For a good comparison of pictorial space and physical space, it is of importance to compare visual directions in the two spaces. Figure 6a shows visual directions for two physical objects—one nearby and the other far away—as function of camera position. Computations were made for the center of the 30 km traffic sign standing at the left side of the road and the mailbox of the distant house in Figure 1a,b. Visual directions of traffic sign and mailbox change by very different amounts between the long-lens (FL 72 mm) and short-lens (FL 36 mm) camera positions. Due to the short object-to-camera distance, visual direction of the traffic sign doubles in eccentricity. Visual direction of the mailbox hardly changes because of the long distance between house and camera. Figure 6b shows the visual directions for the center of the depicted traffic sign and mailbox as function of viewing distance. Due to identical distances between the depicted objects and viewer, visual directions of traffic sign and mailbox change by equal factors between two the viewing distances at which the pictures of Figure 1a,b were taken. Between the 21 cm and 41 cm viewing distances, all visual directions become less eccentric by a factor of two. Visual direction ratio, defined as visual direction of the house divided by visual direction of the traffic sign, was computed for the physical and depicted objects. In physical space, visual direction ratio decreases with more forward positions of the camera because the traffic sign becomes much faster eccentric than the house does (Figure 6c). In pictorial space, visual direction ratio remains constant for viewing distances longer than about 10 cm because the depicted traffic sign and house are equally distant to the viewer (Figure 6d). Visual directions of the physical traffic sign and house at the positions from which the photographs are taken (Figure 6a) determine the values of the ratio. Like for angular size, focal length of the lens does not affect the visual direction ratios because changes of focal length amplify the visual directions of traffic sign and house by identical factors.

## 5. Sizes, Distances, and Distortions in Pictures

**Figure 7:**
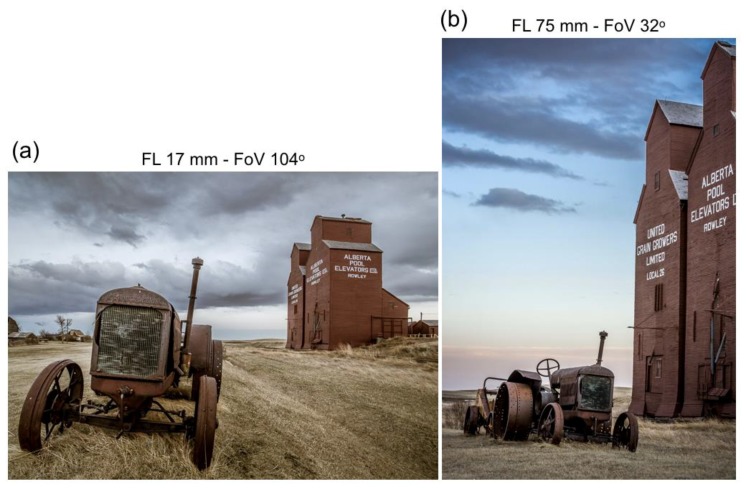
Two photographs of the same perspective scene. Darlene Hildebrandt, a professional photographer working from Edmonton, Alberta, Canada, took both pictures (https://www.digitalphotomentor.com/5-mistakes-beginners-make-using-a-wide-angle-lens-and-how-to-avoid-them/). (**a**) The picture was taken with a full-frame camera equipped with a short lens having a focal length of 17 mm and a field of view of 104°. (**b**) This picture was taken with a long lens (FL 75 mm, FoV 32°). Pictures were used for this study after written consent of the photographer.

**Figure 8:**
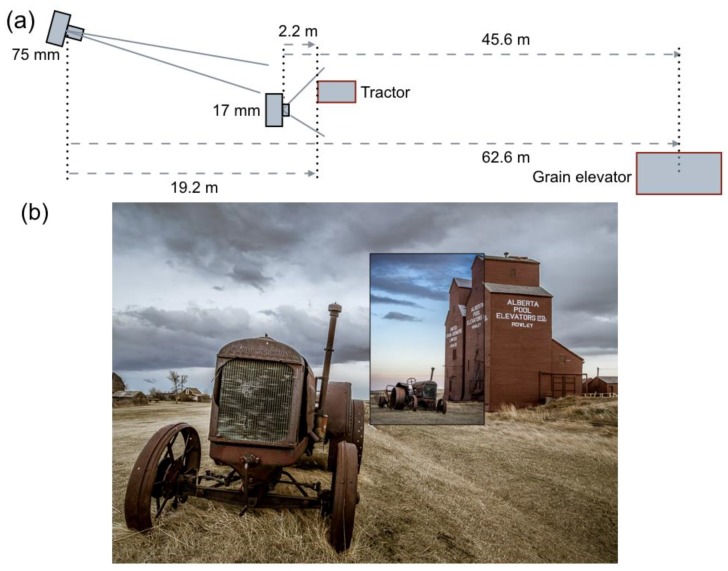
(**a**) Geometry of the physical scene. (**b**) Figure 7a is shown here with Figure 7b on top. Figure 7b has been resized such that the grain elevator is depicted at the same scale in both pictures.

Figure 7 shows two pictures taken by a professional photographer of a tractor and grain elevator. At first sight, sizes and distances of both objects seem incompatible in the two pictures. The picture of Figure 7a was taken with a short lens having a very wide FoV, whereas the picture of Figure 7b was taken with a long lens having a narrow FoV. On the basis of the familiar object sizes and the objects’ positions in the picture, the tractor is seen nearby, and the grain elevator rather far away in the left picture. The grain elevator appears much nearer in the right picture. It is as if the photographer has moved forward. The distance between tractor and elevator seems much shorter in the right picture. If so, then the tractor must have been moved closer to the grain elevator. According to the photographer (see her website), however, the tractor was at the same physical position in both pictures (on the basis of the tractor’s looks, moving it may not be easy). The immobility of the scene, the FoVs and sizes of the pictures, and the physical size of one object make it possible to reconstruct the geometry of the physical scene and the positions from which the pictures were taken (Figure 8a). The track width of 165 cm (65 inch) of the classic Mc Cormick Deering 22–36 tractor served as the familiar size of one physical object. Figure 8a shows that the left picture of Figure 7 is taken from a much shorter distance than the right picture. As a naïve viewer, one has no idea of the different camera positions and settings. As the previous analyses of this study showed, perceived distances are signaled by the angular size of pictured objects. Even knowledge of position and settings of the camera, as the professional photographer may have, does not help in seeing objects at physically correct sizes and distances. Figure 8b shows that resizing the pictures, so that the grain elevators are depicted on the same scale, results in distances of the pictured objects being better in line with physical distances. The tractor on the small picture is seen at a much farther distance than the tractor on the large picture. The grain elevator in the small picture is seen slightly farther away than the grain elevator in the large picture, due to the different camera orientations. The pictorial distances are now qualitatively in accordance with the different camera positions and geometry of the physical scene (Figure 8a).

**Figure 9:**
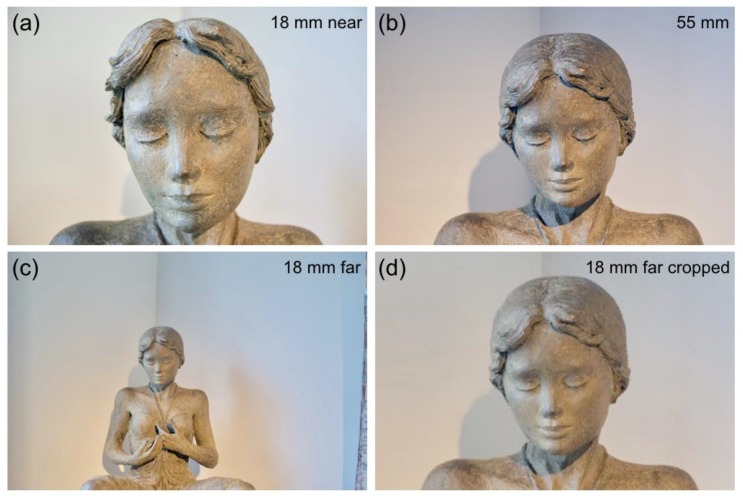
Four pictures of a bronze sculpture of a girl. The camera is fitted with an APS-C sensor. (**a**) Picture taken with an 18 mm lens from a near camera position. (**b**) Picture taken with a 55 mm lens from a far camera position. (**c**) Picture taken with an 18 mm lens from the far camera position of (**b**). (**d**) Picture (**c**), but cropped and expanded to match the size of the face in the other pictures.

The two tractors shown in Figure 8b seem to have different shapes. A conspicuous difference is the angle between the front wheels. In the nearby tractor, the wheels seem to diverge, whereas they seem parallel for the far tractor. The difference is not caused by picture size, because it is also observed between the tractors of Figure 7. Different camera positions caused the difference in angles. The nearby tractor was photographed from a very short distance (Figure 8a). From the distance of 2.2 m, the visual directions of the two wheels differ by 37°. The far tractor was photographed from a distance of 19.2 m (Figure 8a). The visual directions of the front wheels differ by just 5° at that distance. A short-focal-length lens has been used for the nearby tractor, and a long-focal-length lens for the far one. Objects captured with short lenses appear expanded in depth, while those captured with long lenses appear compressed. Figure 1 and the following analysis shows that depth expansion and compression are not related to the length of lenses, but angular size of the depicted objects. Depth compression and expansion can also affect the appearance of a face. Long lenses make a person look smarter, more attractive, and less approachable; short lenses have the opposite effects [59]. The effect of lenses on the appearance of a face was examined by comparing photographs taken with long and short lenses from different distances. Figure 9a shows the face of a bronze statue of a girl photographed with a short lens of 18 mm. Distance between camera and forehead was 0.15 m. Figure 9b shows the face with a longer lens of 55 mm. The distance between camera and forehead was 0.80 m now. The 55 mm lens mounted on an APS-C camera is within the range of focal lengths that is often used for photographing faces. Indeed, the girl’s face looks more natural and attractive. Figure 9c shows, again, the girl photographed with the short lens of 18 mm, but now, with a camera distance of 0.80 m from the forehead. Due to the longer distance, a larger part of the girl than her head is visible on the picture. Figure 9d shows a cropped version of Figure 9c. Comparison of Figure 9b,d shows that the girl’s head looks very similar in both pictures, irrespective of the different lenses used for both pictures. Contrastingly, the girl’s head looks different in Figure 9d than in Figure 9a although the same short lens was used for both pictures. The pictures of Figure 9 show that lenses do not cause facial distortions. It is the distance between camera and face that determines the attractiveness of depicted faces. This is also true for physical faces. The face of the bronze girl looks very similar to the depicted girl of Figure 9a if the viewer is just 0.15 m away from the girl's head. For instance, one cannot see the topside of the head from that position. Due to the three-dimensional shape of the head, a larger portion becomes visible and relative distances between different parts of the face become smaller if one views the statue from a more distant position.

## 6. Distance in Pictorial Space Fitted with Perspective Distance Functions

**Figure 10:**
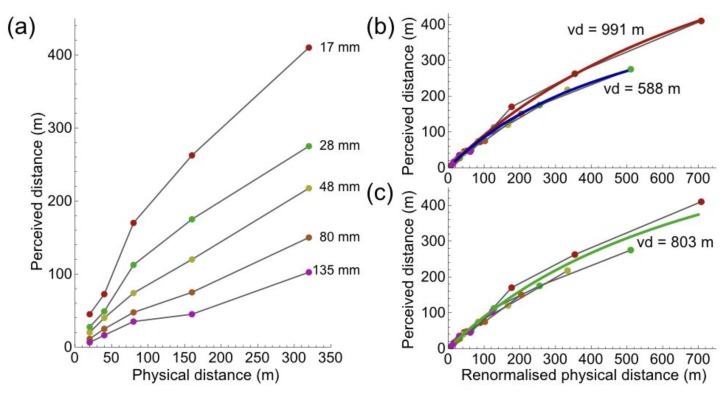
Perceived distance in pictures. (**a**) Data copied from Kraft and Green (1989). (**b**) Data of (**a**) with physical distances renormalized to the FoV of a FL 50 mm lens. Separate fits were computed for perspective distance curves for the 17 mm data (red) and the other data (blue). (**c**) Data of (**b**) fitted with a single perspective distance curve (green).

Until now, the geometry of pictorial space was compared to that of physical space and not visual space. Yet, it has been documented exhaustively in the literature that visual space differs from physical space, in the depth domain. Recent analysis showed that perspective space is a good model of visual space [55]. It is a simple yet powerful model because it describes many experimental results, explains certain visual phenomena, and unifies a number of models of distance perception [56]. To investigate resemblance between distance in pictorial space and visual space, the literature was searched for studies that provided experimental results of depth perceived in pictures. Kraft and Green [57] presented an extensive set of data, which has already been reanalyzed by Cutting [60]. The present finding that angular size is key to distance perception in pictures warrants a third analysis of Kraft and Green’s data. Kraft and Green [57] collected data of 70 observers who judged the distance of objects in pictures. Kraft and Green presented many photographs. Photographs were taken with a full-frame camera and five different lenses: focal lengths of 17, 35, 48, 75, and 135 mm. With such lenses, the horizontal field of view subtends about 105, 60, 45, 32, and 20°, respectively. In two different outdoor environments, Kraft and Green [57] planted poles at distances of 20, 40, 80, 160, and 320 m from a fixed camera. Viewing different arrangements of 50 slides, observers made judgments of the distance of each pole from the camera. Figure 10a shows the graph copied from Kraft and Green [57]. The graph shows that perceived distance depends strongly on the length of the lens. In view of the result of this study, that angular size is the effective stimulus for distance perception, it is relevant to note that Kraft and Green [57] projected all photographs with a Kodak Carousel slide projector on a screen. All pictures had one size, implying that angular sizes of poles in long-lens pictures were too wide relative to those of poles in short-lens pictures. To correct for the different FoVs, physical distances of poles to the camera were renormalized to distances as if the photographs were taken with the FoV of a FL 50 mm lens. Figure 10b shows that the majority of data form a single curve after renormalization. Perceived distances of poles, in the narrow field pictures of 17 mm, deviate from the main curve for the longer physical distances of the poles. The data were fitted with the following distance function for perspective space [56]:

*Perceived distance = vd × Physical distance/(vd + Physical distance)*, where *vd* indicates distance of the vanishing point of perspective space. A derivation of the perceived-distance equation is given in the appendix of [56]. Figure 10b shows two separate fits, one to the 17 mm data and the other fit to the data of the other FLs. Both fits account for more than 99% of the variance. A single fit to all data accounts for just slightly less of the variance, namely 98% (Figure 10c). The excellent fits show that perspective space is a good model for pictorial distance. There is no clear argument that explains the difference between the 17 mm and other FLs in the study of Kraft and Green [57].

## 7. Discussion

The straightforward computations of this study provide insight in the geometry of depicted scenes and the pictorial space that we experience when looking at pictures. Picture size and FoV of the lens are necessary data for the veridical reconstruction of directions and distances of physical objects relative to the camera position. Figure 1 shows traffic signs whose physical distances to the camera (Figure 2) were considerably different in Figure 1a,b. The fact that we perceive the equally large traffic signs at equal depths in both pictures shows that pictorial distances are judged relative to the viewer, rather than camera position. The current study shows that angular size determines the pictorial distance of familiar objects. This conclusion confirms and explains previous research showing that viewers do not compensate for incorrect viewing distance [57,58,61,62,63,64]. FoV of the lens is irrelevant for the computation of pictorial distances. Picture size does neither appear in the computations. The irrelevance of picture size is easily verified by covering a part of a picture in Figure 1 or Figure 5. Picture size only affects perceived distance if it changes the angular size of depicted objects. This occurs when pictures are uniformly compressed or enlarged. Angular size is the effective stimulus for perceived size and distance in the perspective space model [56]. The model proved to be a successful model for the visual perception of sizes, distances, and angles of physical objects at both near and far distances [34,53,54,55,56,65]. Reanalysis of the data of Kraft and Green [57], such that the perceived distances of depicted objects were related to angular size, shows that perspective space is also a good model for far pictorial distances. The distances of vanishing point of the model fits shown in Figure 10b,c may not represent the real vanishing distance of pictorial space, because it is not clear from Kraft and Green’s paper whether normalization of the data to the FL 50 mm format produced the correct angular sizes. Normalization may have produced angular sizes times a magnification factor.

### 7.1. Invariance in Picture Perception

The visual scene changes considerably if an observer moves forward in a physical environment with nearby and far objects. Adjacent objects approach faster and move outward in front of far objects, until they disappear out of sight. Relative distances, directions, and sizes remain only constant if the observer stands still. Scene dynamics are very different if you move towards a picture of the same environment. Although all objects move outward and get closer, the scene itself is frozen. Absolute distances, directions, and sizes of the depicted objects depend on viewing distance. However, as this study shows, relative distances, directions, and sizes remain constant and are associated with standstill in a real three-dimensional environment. The relationship with immobility may explain the invariance of pictorial scenes if these are viewed at incorrect distances. Scenes are considered trustworthy representations of physical scenes over a long range of viewing distances before they seem compressed or expanded in extreme conditions. Estate agents exploit the invariance by presenting rooms wider than they really are in their advertisements. Perceptual invariance across viewing position has been of interest to many visual scientists. Most studies investigated the shape of pictorial objects from oblique viewing positions [2,7,11,12,23,51,52,66,67]. Effects of viewing distance on perceived size, distance, and direction have received less attention [58,61]. Apparently, perceptual invariance as a function of viewing distance has usually been taken for granted.

### 7.2. Misconceptions about Picture Perception

There exists a widely held erroneous belief in the literature of picture perception. The belief is that pictures viewed from positions other than the camera position are valid two-dimensional representations of physical space. The consequence of the belief is that perceived distances and directions obtained from such pictures can be used to draw conclusions about the geometry of pictorial space. Although the belief is usually not expressed explicitly, there are various examples of incorrect conclusions. One incorrect conclusion involves pictorial distortions. At a certain viewing distance, called the correct viewing distance, a picture induces the same retinal image as the physical scene. A picture is viewed from the correct distance if the angular size of the picture equals the FoV of the lens. Changing the size of a picture by compression or expansion changes the correct viewing distance too. Figure 1, Figure 3 and Figure 4 illustrate this. Compression of Figure 1b,c changed the correct viewing distance from 41 cm (Figure 3b) to 22 cm (Figure 4b). Viewing the picture from an incorrect distance magnifies the angular sizes of all depicted objects by a common factor (Figure 5d). Compression or expansion of the picture causes the same effect. Such changes in angular size are very different from those associated with moving forward or backward in the physical environment. Then, near objects change much more in angular size than far objects do (Figure 5a). Due to the angular size–distance relationship, pictorial distances behave differently as function of viewing distance, than visual distances as a function of camera position (compare Figure 3 and Figure 4 with Figure 2). A widely held opinion is that photographs of scenes captured with short-focal-length lenses appear expanded in depth, while those captured with long lenses appear compressed [58]. The examples presented in Figure 1, Figure 8, and Figure 9 show that expansion or compression of depth is not related to focal length, but to the angular sizes of near and far objects in the picture. The ratio between angular sizes of depicted objects depends on the camera position from which the picture is taken, not on focal length of the lens. Each ratio is unique for a certain camera position (Figure 5c), implying that the picture could not have been made from any other camera position. From an incorrect viewing distance, the picture is a projection of a non-existing physical scene. Therefore, it is inappropriate to call depth expansion or compression distortions because, from incorrect viewing distances, they reflect the correct perspective projection of non-existing physical scenes. A related misconception concerns the distortion of faces by short-focal-length lenses [58,61]. Short-focal-length lenses do not expand depth if pictures are viewed at the correct distance. The example of the bronze girl in Figure 9c,d shows that such lenses not necessarily exaggerate the depth of a face. Facial distortions occur when pictures that were taken from extremely close camera positions are viewed from longer distances. Another incorrect conclusion involves pictorial direction. Koenderink and colleagues [68] have argued that visual directions are parallel. Evidence came, among others, from perceptual judgments of the people’s orientations while these were seated on chairs next to each other with ample space between them. Naïve observers made the judgments from pictures taken by a camera equipped with wide-angle lenses (horizontal FoV 104°). A linear-perspective picture showed the persons, i.e., the authors, in a fronto-parallel row. The authors’ orientations were judged as rotated with respect to the straight-ahead direction. An equiangular projection showed the authors in a circular (about the camera) row, all facing the camera. Now the authors were judged as fronto-parallel, and seated in strict military order [68]. The conclusion that visual directions are parallel denies the fact that the visual and pictorial spaces are perspective spaces. The tractor of Figure 7a provides a good illustration. The left front wheel of the tractor is oriented to the camera and positioned close to the center of the picture. Figure 7b shows that the front wheels of the tractor are parallel to each other in physical space. Therefore, both wheels are aligned with the straight-ahead direction of the camera in Figure 7a. At the off-center position of the right front wheel, the wheel is not oriented to the camera in physical space. If we would look directly at the right front wheel from the same physical position, we would see it at an angle too. To give a counterexample in Koenderink’s military terminology: soldiers standing off-center in a platoon do not appear to look at their commander. They better not! This leaves, unanswered, the question why naïve observers judged the facing authors as fronto-parallel. The answer is probably related to the fact that judgments were made from pictures, and the observation that wide-angle pictures are usually viewed from too far [58]. The tractor of Figure 7a is again a good illustration. From the geometrical data of Figure 8a and the known track width of the front wheels, it was computed that the physical right front wheel made an angle of 37° with its direction to the camera. Prolonging the viewing distance or uniformly compressing a picture leaves the shape of depicted objects unchanged. However, if the picture is viewed from 20 cm in front of the left front wheel, the visual angle to the right front wheel is only 17° instead of 37°. As a result, the right front wheel will appear rotated outward by 20°. The generalization of this example is that angles in short-focal-lens pictures appear usually different from the physical angles.

## 8. Conclusions

Computations made on different pictures of one perspective scene reveal that angular size is the effective stimulus for the perceived distance of objects in pictures. Although pictorial distances and directions of object change as function of viewing distance, ratios of distances and directions are constant. Pictorial distances and directions were computed from pictures by using the rules that predict visual distances and directions of physical objects. Data of distance judgments obtained from the literature shows that perspective space is as good a model for pictorial space as it is for visual space. The derived pictorial geometry reveals a few misconceptions about picture perception.
